# Two-year outcomes of sleeve gastrectomy versus gastric bypass: first report based on Tehran obesity treatment study (TOTS)

**DOI:** 10.1186/s12893-020-00819-3

**Published:** 2020-07-20

**Authors:** Alireza Khalaj, Erfan Tasdighi, Farhad Hosseinpanah, Maryam Mahdavi, Majid Valizadeh, Elham Farahmand, Hamidreza Taheri, Maryam Barzin

**Affiliations:** 1 Tehran Obesity Treatment Center, Department of Surgery, Faculty of Medicine, Shahed University, Tehran, Iran; 2 Obesity Research Center, Research Institute for Endocrine Sciences, Shahid Beheshti University of Medical Sciences, Tehran, Iran

**Keywords:** Bariatric surgery, Sleeve gastrectomy, Gastric bypass, Safety, Efficacy, Outcomes, Report

## Abstract

**Background:**

Bariatric surgery is an effective treatment for obesity and its associated comorbidities. This is the first comprehensive report of a prospective cohort study, comparing sleeve gastrectomy (SG) with gastric bypass (GB) regarding their effectiveness and safety.

**Methods:**

The prospectively collected data of patients, who presented to a specialized bariatric center and underwent a primary bariatric procedure, were compared in terms of weight loss, remission of obesity-associated comorbidities, complication rate, and quality of life improvement at 6-, 12-, and 24-month follow-ups.

**Results:**

Of 3287 patients (78.6% female) analyzed, 67% (*n* = 2202) and 33% (*n* = 1085) underwent SG and GB, respectively. Effective outcomes were reported in both groups regarding the body composition indices. Type 2 diabetes mellitus (T2DM) remission rate at the end of follow-up was 53.3% and 63.8% in the SG and GB groups, respectively. Following the propensity score-adjusted analysis, the T2DM remission rate was not significantly different between the groups. Conversely, the remission rate of hypertension in the 24-month follow-up (39.1% vs. 54.7%) and the remission rate of dyslipidemia in all follow-ups were lower in the SG group, compared to the GB group. Moreover, both procedures caused substantial improvements in various domains of quality of life. The surgery duration, early complication rate, and nutritional deficiencies were lower in the SG group, compared to the GB group.

**Conclusion:**

Both surgical procedures were effective in the control of obesity and remission of its comorbidities. However, since SG was associated with a lower rate of complications, it seems that SG should be considered as a suitable procedure for obese patients, especially those with a healthier metabolic profile.

## Background

Evidence shows that bariatric surgery is more effective than conventional therapy for the control of obesity and its associated comorbidities [1]. Although bariatric surgery has been performed extensively for several decades around the world [2], there is no consensus with regard to the safety and efficacy of different procedures [3, 4]. According to recent estimates, sleeve gastrectomy (SG) is currently the most common procedure around the world (46% of all bariatric procedures), followed by Roux-en-Y gastric bypass (RYGB) (40% of all procedures) [3].

The results of meta-analyses comparing SG with RYGB are controversial [5, 6]. The first meta-analysis in this area showed that SG and gastric bypass (GB) were not significantly different regarding excess weight loss (EWL%) 18 months after surgery, whereas the resolution of T2DM was better in GB. Conversely, another study showed a higher EWL% in GB patients, while no significant difference was found regarding T2DM remission. Moreover, two randomized clinical trials (RCTs) comparing SG with RYGB reported that these surgeries did not differ in terms of EWL% [7, 8]. However, a recent RCT showed that GB is a superior procedure regarding weight loss and diabetes remission [9].

Not only the effectiveness of bariatric surgery can vary with procedure, but also the outcomes of bariatric surgery vary between populations from different geographical regions [10]. Nevertheless, the published data are inconclusive in this area, and most studies have been conducted in North America, Europe, and East Asia. The present study is the first comprehensive report of a prospective cohort study evaluating a broad spectrum of outcomes in a two-year follow-up in the Middle East (MENA) regions.

## Materials and methods

### Study protocol

Tehran Obesity Treatment Study (TOTS) is a prospective cohort study of morbidly obese patients, presenting to our specialized treatment center to undergo bariatric surgery, as described in detail elsewhere [11]. In this report, we reviewed all cases in the TOTS database between March 1, 2013 and February 31, 2017, based on complete two-year follow-up data. The patients were divided into two groups, including 2202 patients who underwent laparoscopic SG and 1085 patients who underwent laparoscopic GB (160 underwent RYGB and 925 underwent one-anastomosis gastric bypass [OAGB]). The follow-up rate was 86, 91, and 64% at 6, 12, and 24 months after surgery, respectively, which was not significantly different between the SG and GB groups.

### Surgical procedures

Patients, who had no history of bariatric surgery, underwent primary SG or GB. A single surgical team performed all operations with a standard five-port laparoscopic approach under general anesthesia. SG was performed over a 36-F bougie and reinforced with an omental pouch. On the other hand, GB was performed as either RYGB or OAGB. RYGB involves the construction of a small gastric pouch and anastomosis to the antecolic Roux limb of the jejunum (150 cm), in addition to side-to-side jejunojejunostomy at the biliopancreatic limb (50 cm). OAGB is a modification of standard RYGB, which uses a long gastric tube with an antecolic loop gastrojejunostomy. In this approach, a long gastric tube is created using an Endo GIA™ stapler from the incisura angularis to the angle of His over a 36-F bougie. A loop gastroenterostomy is also performed 160–200 cm distal to the ligament of Treitz with an Endo-GIA™ stapler.

### Measurements

Trained investigators collected the required data according to the study protocol [11]. Presurgical data, including demographic characteristics, anthropometric indices, comorbidities, and blood test results, were obtained. Anthropometrics included weight, height and waist circumference measurements according to WHO guidelines. Body composition was assessed using the portable bioelectrical impedance analyzer (InBody 370, Biospace, Seoul, Korea). Participants were asked to comply with the following criteria prior to impedance analysis: fasting overnight or for a minimum of 4–5 h, no exercise for at least 12 h, no alcohol for at least 24 h, balanced hydration, and lying in a supine position for at least 5 min prior to examination. Resistance to the alternating current flow (500- μA at 50/60 kHz) was measured with the patient standing on the analyzer’s platform and interpreted using the “standard” option of the manufacturer’s software. Fat mass (FM, in kg), fat-free mass (FFM, in kg) and percent body fat mass (%FM) were obtained.

Fasting plasma glucose (FPG), serum triglyceride (TG), total cholesterol (TC) based on enzymatic colorimetric method, and high-density lipoprotein-cholesterol (HDL-C) after precipitation of apolipoprotein B-containing lipoproteins with phosphotungstic acid were determined, using relevant kits. All samples were analyzed when the internal quality control met the acceptable criteria. The inter- and intra-assay coefficients of variations at baseline were both 2.2% for FPG, 2 and 0.5% for HDL-C, and 1.6 and 0.6% for TG, respectively. The micronutrient status and serum concentrations of vitamins (B12 and D), minerals (calcium, phosphorus, copper, and zinc), hemoglobin, hematocrit, and iron profiles (total iron binding capacity [TIBC], iron, and ferritin) were assessed, using routine blood samples collected before surgery and during follow-ups.

The serum levels of vitamin B12 and D were measured using chemiluminescent immunoassay and enzyme immunoassay, respectively. Calcium and phosphorus levels were also measured based on methyl thymol blue colorimetry and UV-endpoint phosphomolybdate method, respectively. Moreover, copper and zinc were measured by colorimetric methods using 3,5-dibromo-2-paridylase and 5-bromo-2-paridylase, respectively. Additionally, serum hemoglobin and ferritin levels were measured using the cyanmethemoglobin method and human ferritin enzyme immunoassay, respectively. Finally, serum iron and TIBC concentrations were assessed using spectrophotometric and colorimetric methods. The reference values are summarized in Table 1 in the Supplementary Appendix.

### Outcome definitions

#### Weight loss

For evaluating weight loss, body mass index change (∆BMI), percentage of total weight loss, and percentage of excess weight loss (EWL%) were calculated as follows:

$$ \varDelta \mathrm{BMI}=\mathrm{Postop}\ \mathrm{BMI}\hbox{-} \mathrm{Initial}\ \mathrm{BMI} $$

$$ \mathrm{TWL}\%=\left[\left(\mathrm{Initial}\kern0.17em \mathrm{weight}\right)-\left(\mathrm{Postop}\kern0.17em \mathrm{weight}\right)\right]/\left[\left(\mathrm{Initial}\kern0.17em \mathrm{weight}\right)\right]\times 100 $$

$$ \mathrm{EWL}\%=\left[\left(\mathrm{Initial}\kern0.17em \mathrm{weight}\right)-\left(\mathrm{Postop}\kern0.17em \mathrm{weight}\right)\right]/\left[\left(\mathrm{Initial}\kern0.17em \mathrm{weight}\right)-\left(\mathrm{Ideal}\kern0.17em \mathrm{weight}\right)\right]\times 100 $$

where the ideal weight is defined by the weight corresponding to a BMI of 25 kg/m^2^.

#### Obesity-associated comorbidities

Three major obesity-associated comorbidities, i.e., type 2 diabetes mellitus (T2DM), hypertension (HTN), and dyslipidemia, were assessed. Each comorbidity was followed-up according to standardized outcome reporting in metabolic and bariatric surgery (Table 2 in the Supplementary Appendix) [12].

#### Complications

Major complications were defined as those requiring the patient’s return to the operating room, prolonged hospital stay beyond 7 days, and need for re-admission. All other complications were regarded as minor. Our primary endpoints in this study were early (< 30 days) and late (> 30 days) complications, major and minor complications, length of hospital stay, and surgery duration.

#### Quality of life

Quality of life was assessed using the Iranian version of Short-Form Health Survey (SF-36), which measures eight health-related components, including physical, mental, and social aspects of health [13].

### Follow-up and postoperative care

Following surgery, the patients, irrespective of their treatment group, underwent a strict post-op protocol. Each patient underwent comprehensive assessments by the medical team at 1, 3, 6, 12, and 24 months after surgery to make sure that they adhere to the protocol. Our post-op care team included an obesity expert, a nutritionist, and a sport and exercise medicine physician. Patients of both groups received a similar calorie-restricted diet (10–35% protein) and were prescribed vitamin and mineral supplements daily up to 6 months. The SG patients continued their diet based on their individual clinical and biochemical assessments. Moreover, all patients followed a physical activity program (combined aerobic-resistance activities) at least 30 min per day postoperatively.

### Statistical analysis

Normally distributed continuous variables were expressed as mean ± SD, and skewed continuous variables were expressed as median and interquartile range (IQR 25–75%). Categorical variables were also reported as frequency (percentage). Normally distributed variables were analyzed using two-tailed independent sample *t*-test, while variables with a skewed distribution were analyzed using Mann–Whitney test. Qualitative variables were analyzed using Chi-square and Fisher’s exact tests, when appropriate. Moreover, a propensity score (PS)–based method was used to control for confounding factors by balancing the distribution of confounders for the surgery type.

In addition, factors influencing the choice of surgery, outcomes of T2DM, HTN, and dyslipidemia remission were identified. A logistic regression model was used to estimate the probability of treatment (or PS) with surgery type as the outcome, adjusted for sex, age, and baseline BMI as the outcomes of all comorbidities; FPG, hemoglobin A1C (HbA1C), duration of DM, and insulin therapy as the outcomes of T2DM; diastolic blood pressure (DBP) and systolic blood pressure (SBP) as the outcomes of HTN; and LDL, HDL, TG, and cholesterol as the outcomes of dyslipidemia. Generally, inverse probability of treatment weighting (IPTW) uses PS as weights to create a dummy sample in which the distribution of covariates is independent of surgery type. IPTW was calculated as 1/PS for those who underwent GB and as 1/(1 − PS) for those who underwent SG. All analyses were performed in SPSS Version 20 (SPSS, Chicago, IL, USA). Two-tailed *P*-values less than 0.05 were considered statistically significant.

## Results

A total of 3287 patients (78.6% female) were included in the analysis. Overall, 2202 patients undergoing SG, with the mean age of 38.0 ± 11.8 years and the mean BMI of 44.6 ± 5.7 kg/m^2^, were compared with 1085 patients undergoing GB, with the mean age of 39.4 ± 10.7 years and the mean BMI of 45.5 ± 6.1 kg/m^2^. The baseline characteristics of the patients are shown in Table 1. There was no significant difference between the two surgery groups regarding metabolic indices, except for FPG, HbA1c%, and LDL-C. Patients in the SG group had lower FPG (105.8 ± 28.5 vs. 117.9 ± 49.9, *P* < 0.001) and HbA1c%, compared to the GB group. The prevalence of T2DM and insulin therapy were significantly lower in the SG group, compared to the GB group (24.1 vs. 35.9%, *P* < 0.001; 8.7 vs. 22.9%, *P* < 0.001, respectively). Based on the findings, the serum level of LDL-C was higher in the SG group, compared to the GB group (112.3 ± 31.5 vs. 109.5 ± 32.2, *P* = 0.023).

**Table 1:** Baseline characteristic of patients underwent SG or GB bariatric surgery

	SG (*n* = 2202)	GB (*n* = 1085)	*P*-value
Sex female,	1683 (76.4)	903 (83.2)	**< 0.001**
Age, year,	38.0 ± 11.8	39.4 ± 10.7	**.002**
Age groups, n (%)			**0.001**
< 40	1264 (57.8)	561 (51.9)	
≥ 40	922 (42.2%)	519 (48.1%)	
Fat mass	59.7 ± 11.6	60.7 ± 11.6	**0.020**
Fat percent	49.6 ± 4.8	50.1 ± 4.8	**0.004**
Smoking status			.**031**
Never smokers	1808 (87.2)	916 (90.4)	
Current smokers	164 (7.9)	59 (5.8)	
Anthropometric indices			
BMI, kg/m^2^	44.6 ± 5.7	45.5 ± 6.1	**< 0.001**
BMI groups			**< 0.001**
BMI < 45 kg/m^2^	1306 (59.6)	557 (51.4)	
BMI ≥45 kg/m^2^	885 (40.4)	526 (48.6)	
Weight, kg	121.0 ± 21.0	121.4 ± 20.0	.596
Height, cm	164.5 ± 9.4	163.1 ± 8.8	**< 0.001**
Waist circumference, cm	123.2 ± 15.1	124.3 ± 14.3	.061
Metabolic indices			
SBP (mmHg)	123.6 ± 12.9	123.4 ± 12.1	.776
DBP (mmHg)	79.7 ± 8.1	79.4 ± 7.4	.207
FPG (mg/dL)	105.8 ± 28.5	117.9 ± 49.9	**< 0.001**
HbA1c%	5.5 (5.1–6.0)	5.6 (5.2–6.4)	**< 0.001**
Triglyceride, mg/dL	140 (103–187)	143 (107–195)	.103
HDL, mg/dL	47.4 ± 12.0	47.0 ± 11.3	.423
LDL, mg/dL	112.3 ± 31.5	109.5 ± 32.2	.**023**
Total cholesterol, mg/dL	190.8 ± 37.7	189.4 ± 39.6	.370
Creatinine, mg/dL	.92 ± .39	.92 ± .41	.899
AST (U/L)	23.3 ± 13.6	22.9 ± 13.7	.452
ALT (U/L)	31.1 ± 23.1	29.8 ± 24.2	.121
Medication			
Insulin	31 (8.7)	69 (22.9)	**< 0.001**
Oral glycemic medicines	206 (58.1)	175 (58.2)	.329
Dyslipidemia medicines	448 (20.3)	307 (28.3)	**< 0.001**
Blood pressure medicines	469 (21.3)	278 (25.6)	**.006**
Comorbidities			
Diabetes	493 (24.1)	376 (35.9)	**< 0.001**
Hypertension	622 (30.2)	329 (32.0)	.321
Dyslipidemia	1820 (85.5)	926 (87.0)	.256

*SG* sleeve gastrectomy, *GB* gastric bypass, *WC* waist circumference, *BMI* body mass index, *SBP* systolic blood pressure, *DBP* diastolic blood pressure, *FPG* fasting plasma glucose, *TG* triglyceride, *HDL* high-density lipoprotein, *ALT* alanine transaminase, *AST* aspartate transaminase

Data are presented as mean ± SD or n (%) expect Triglyceride and HbA1c which are presented as median (IQ 25–75)

### Anthropometric and body composition indices

**Fig. 1:**
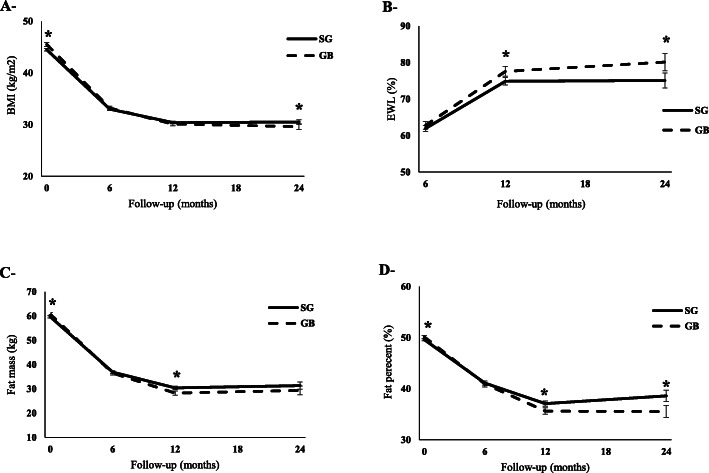
The anthropometric and body composition indices over time: **a** BMI (kg/m^2^); **b** EWL (%); **c** fat mass (kg); and **d** fat percentage (%)

Changes in the anthropometric and body composition indices in the two-year follow-up are shown in Fig. 1. All anthropometric indices in the two groups improved significantly after surgery (*P*_trend_ < 0.001), although it was more significant during the first 6 months. However, BMI was not significantly different between the two groups, except in the 24-month follow-up, when the SG group had a higher BMI than the GB group (Fig. 1a). Based on the results, EWL% was 61.9 ± 15.7, 74.8 ± 19.1, and 75.0 ± 21.9 in the SG group and 62.7 ± 15.3, 77.5 ± 18.4, and 80.1 ± 20.8 in the GB group at 6-, 12-, and 24-month follow-ups, respectively. EWL% was lower in the SG group, compared to the GB group in 12- and 24-month follow-ups (*P* = 0.002 for both) (Fig. 1b).

Despite the lower fat mass in the SG group compared to the GB group at baseline (59.7 ± 11.6 vs. 60.7 ± 11.6, *P* = 0.020), it was higher in the SG group at 12 months after surgery (30.3 ± 9.7 vs. 28.2 ± 9.8, *P* < 0.001) (Fig. 1c). Changes in fat percentage and its comparison between the groups showed the same trend during the follow-ups (Fig. 1d). Moreover, ΔBMI and TWL% were more prominent in the GB group in all follow-ups (Figure 1A and Figure 1B in the Supplementary Appendix).

### Metabolic indices

**Fig. 2:**
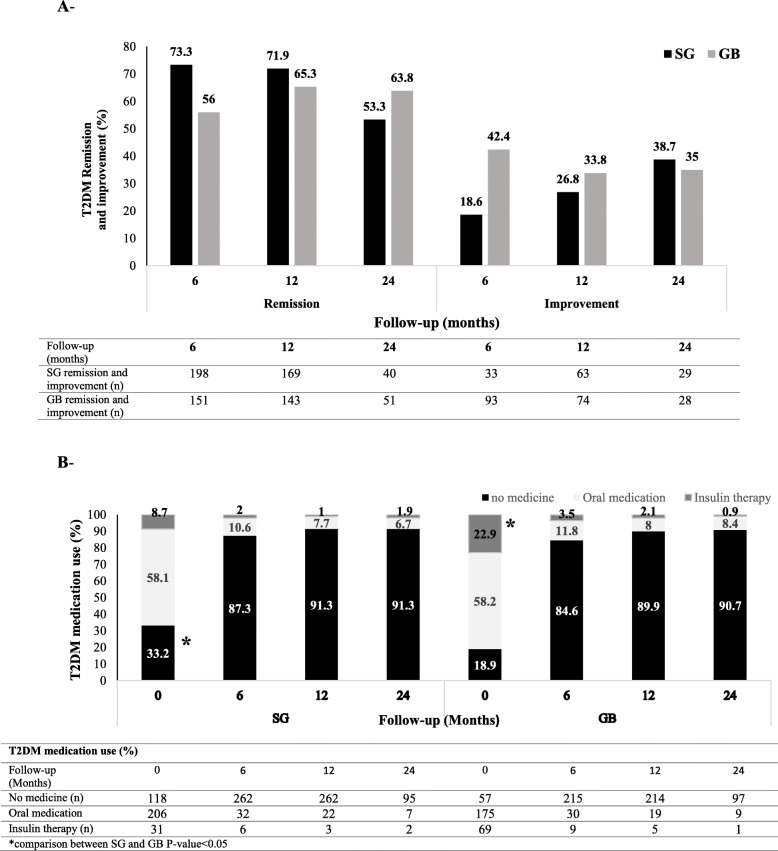
Diabetes remission, improvement, and medication use: **a** Proportion of patients with diabetes remission and improvement; and **b** diabetes medications and the mean number of used glucose-lowering drugs

Changes in metabolic indices during the follow-ups are shown in Figure 2 in the Supplementary Appendix. FBS and HbA1c% decreased significantly after surgery in both groups (*P*_trend_ < 0.001), (Figure 2A and Figure 2B in the Supplementary Appendix). The GB group showed more significant results regarding TC and LDL reduction in all follow-ups (Figure 2C and Figure 2D in the Supplementary Appendix). On the other hand, HDL increased in both groups, although the SG group showed higher levels of HDL throughout the follow-up (Figure 2F in the Supplementary Appendix). SBP and DBP were not significantly different between the groups at baseline or during the follow-ups (Figure 2G and Figure 2H in the Supplementary Appendix). They only decreased significantly during the first 6 months after surgery in both groups (*P* < 0.001).

### Obesity-associated comorbidities

#### T2DM

The prevalence of T2DM at baseline was 439 (24.1%) and 376 (35.9%) in the SG and GB groups, respectively, which was significantly higher in the GB group (*P* < 0.001). Of all patients with DM, 671 cases were followed-up for 2 years, and DM remission and medication reduction were evaluated in these individuals. Out of 671 patients, 364 (54.2%) and 307 (45.8%) underwent SG and GB, respectively. The results showed that T2DM duration, mean FPG, and HbA1c% were significantly lower in the SG group, compared to the GB group.

At baseline, 118 (33.2%) and 57 (18.9%) patients did not use any DM medications, while 31 (8.7%) and 69 (22.9%) patients required insulin alone or in combination with other drugs, respectively, which was significantly lower in the SG group, compared to the GB group (*P* < 0.001, Fig. 2). Insulin therapy significantly reduced in both groups during the follow-up, and it was found to be lower than 2% in the 24-month follow-up. The number of patients with no DM medications in the SG group versus the GB group was 262 (87.3%) versus 215 (84.6%), 214 (89.9%) versus 262 (91.3%), and 95 (91.3%) versus 97 (90.7%) at 6-, 12-, and 24-month follow-ups, respectively (Fig. 2b).

The rate of T2DM remission was 73.3, 71.9, and 53.3% in the SG group and 56, 65.3, and 63.8% in the GB group at 6-, 12-, and 24-month follow-ups, respectively. Additionally, T2DM improvement was reported in 18.6, 26.8, and 38.7% of subjects in the SG group and 42.4, 33.8, and 35% of subjects in the GB group at 6-, 12-, and 24-month follow-ups, respectively (Fig. 2a). After PS-adjusted multivariable analysis, the surgical techniques were found to be similar regarding T2DM remission at all three time points of the follow-up (Table 2).

**Table 2:** Odds ratios (OR) for remission of obesity-associated comorbidities and its components between surgery groups (SG vs.GB) at 6-, 12- and 24-months follow-up

Obesity-comorbidities	Follow up (months)	Propensity score-adjusted OR	95% CI	***P*** value
**T2DM remission**	**6**	0.82	0.51–1.31	0.412
	**12**	1.15	0.69–1.91	0.574
	**24**	2.00	0.88–4.54	0.096
**HTN remission**	**6**	0.79	0.56–1.10	0.169
	**12**	0.96	0.68–1.36	0.850
	**24**	2.10	1.22–3.60	**0.007**
**Dyslipidemia remission**	**6**	1.30	1.00–1.69	**0.044**
	**12**	1.86	1.47–2.35	**0.000**
	**24**	2.59	1.58–4.24	**0.000**

*T2DM* Type 2 diabetes mellitus, *HTN* hypertension

#### HTN

At baseline, 622 (30.2%) subjects in the SG group and 329 (32.0%) subjects in the GB group had HTN, which was not significantly different between the two groups. Of all patients with HTN (*n* = 734), 466 (63.4%) patients undergoing SG and 268 (36.6%) patients undergoing GB were followed-up for 2 years, and HTN remission and reduction of anti-HTN medication use were evaluated in these individuals.

**Fig. 3:**
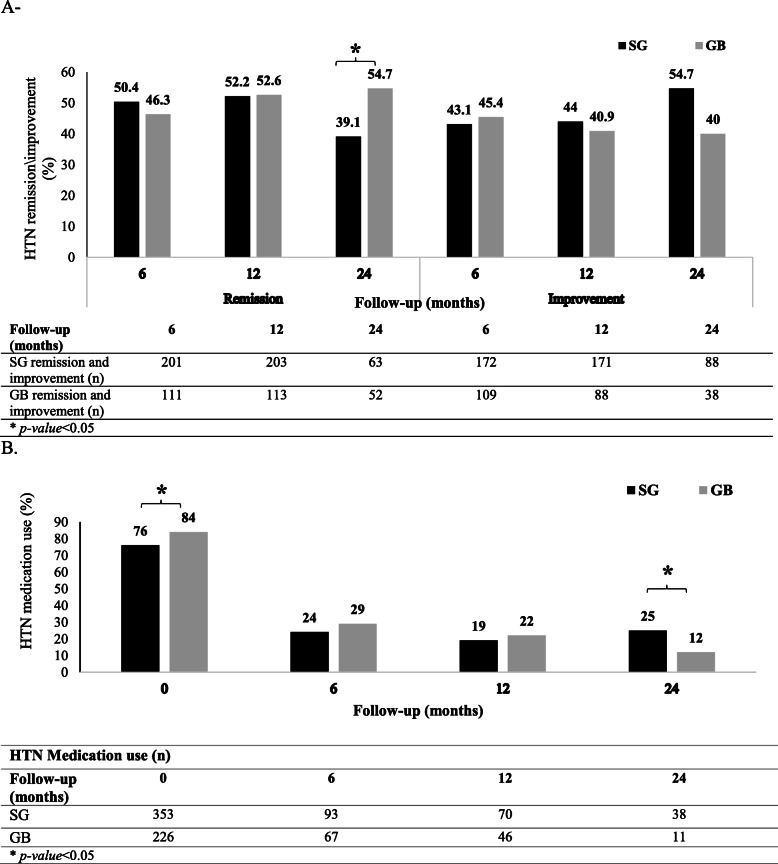
HTN remission, improvement, and medication use: **a** Proportion of patients with HTN remission and improvement; and **b** HTN medications and the mean number of used blood pressure-lowering drugs

At baseline, 353 (75.7%) and 226 (84.3%) patients used HTN medications in the SG and GB groups, respectively (Fig. 3). Medication use significantly reduced in both groups after surgery. The number of patients using anti-HTN medications in the SG group versus the GB group was 93 (23.7%) versus 67 (29.0%), 70 (18.7%) versus 46 (21.9%), and 38 (24.8%) versus 11 (11.9%) at 6-, 12-, and 24-month follow-ups, respectively. Medication use was higher in the SG group, compared to the GB group only in the 24-month follow-up (*P* = 0.010) (Fig. 3b).

The prevalence of HTN remission was 50.4, 52.2, and 39.1% in the SG group and 46.3, 52.6, and 54.7% in the GB group at 6-, 12-, and 24-month follow-ups, respectively. HTN improvement was estimated at 43.1, 44, and 54.7% in the SG group and 45.4, 40.9, and 40.0% in the GB group at 6-, 12-, and 24-month follow-ups, respectively (Fig. 3a). After PS-adjusted multivariable analysis, the GB group showed a higher rate of HTN remission, compared to the SG group only in the 24-month follow-up (OR = 2.10, 95% CI: 1.22–3.60, *P* = 0.007) (Table 2).

#### Dyslipidemia

The prevalence of dyslipidemia at baseline was 1820 (85.5%) and 926 (87.0%) in the SG and GB groups, respectively, which was not significantly different between the groups. Of all patients with dyslipidemia, 2116 cases were followed-up for 2 years, and dyslipidemia remission and medication use reduction were evaluated in these individuals. Out of 2116 patients, 1349 (63.7%) underwent SG, and 767 (36.3%) underwent GB.

**Fig. 4:**
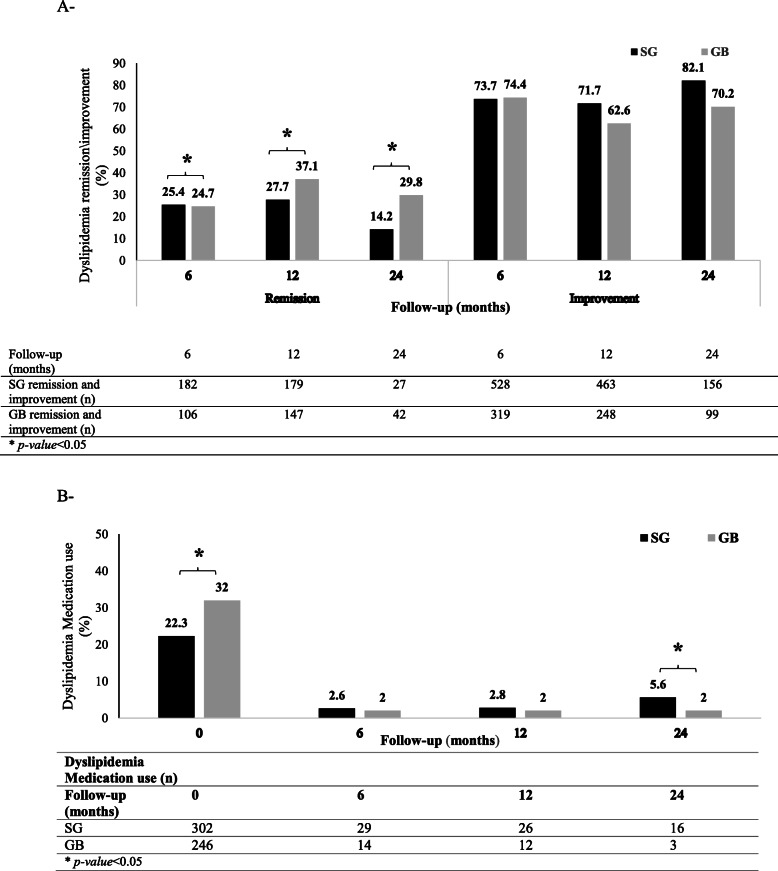
Dyslipidemia remission, improvement, and medication use: **a** Proportion of patients with dyslipidemia remission and improvement; and **b** dyslipidemia medications and the mean number of used lipid-lowering drugs

At baseline, 302 (22.3%) and 246 (32.0%) patients used dyslipidemia medications in the SG and GB groups, respectively (Fig. 4). Medication use significantly reduced in both groups after surgery. The number of patients using dyslipidemia medications in the SG group versus the GB group was 29 (2.6%) versus 14 (2.2%), 26 (2.8%) versus 12 (2.0%), and 16 (5.6%) versus 3 (1.5%) at 6-, 12-, and 24-month follow-ups, respectively. Medication use was higher in the SG group, compared to the GB group only in the 24-month follow-up (*P* = 0.022) (Fig. 4b).

The prevalence of dyslipidemia remission was 25.4, 27.7, and 14.2% in the SG group and 24.7, 37.1, and 29.8% in the GB group at 6-, 12-, and 24-month follow-ups, respectively. Based on the findings, dyslipidemia improvement was estimated at 73.7, 71.7, and 82.1% in the SG group and 74.4, 62.6, and 70.2% in the GB group at 6-, 12-, and 24-month follow-ups, respectively (Fig. 4a). Based on the PS-adjusted multivariable analysis, the GB group had a significantly higher rate of dyslipidemia remission, compared to the SG group in all follow-ups (Table 2).

#### Nutritional deficiencies

The prevalence of all micronutrient deficiencies at baseline and follow-ups is described in Table 3. The most common deficient micronutrients in both SG and GB groups were vitamin D, vitamin B12, hemoglobin, and serum iron. In general, nutritional deficiencies were less prevalent in the SG group, compared to the GB group. Hemoglobin and hematocrit deficiencies were significantly lower in the SG group, compared to the GB group in all follow-ups. Moreover, in the 12-month follow-up, iron (5.2% vs. 12.2%), ferritin (15.7% vs. 25.0%), copper (5.8% vs. 8.5%), and calcium (0.6% vs. 3.7%) deficiencies were significantly less prevalent in the SG group, compared to the GB group. There was no case of hypoalbuminemia in the SG group, whereas in the GB group, its prevalence was 1.5 and 1.0% at 12- and 24-month follow-ups, respectively.

**Table 3:** Nutritional deficiencies in each surgery group pre-operation and during follow-up

Nutritional deficiencies	Surgical technique	Pre operation	Post operation		
			12th month	24th month	***P***-value
Iron deficiency Anemia	**SG**	326(15.3)	202 (25.1)	41 (27.7)	<.001
	**GB**	199 (18.8)	197 (39.6)	88 (55.0)	<.001
	*P*-value	.014	<.001	<.001	–
Hematocrit, deficiency	**SG**	159 (7.7)	134(16.7)	28 (18.9)	<.001
	**GB**	101 (10.0)	148 (29.8)	67 (42.1)	<.001
	*P*-value	.034	<.001	<.001	
Ferritin deficiency	**SG**	105 (6.9)	91 (15.7)	22 (25.6)	<.001
	**GB**	69 (8.5)	99 (25.0)	56 (45.9)	<.001
	*P*-value	.163	<.001	.003	–
Iron deficiency	**SG**	164 (8.7)	32 (5.2)	12 (14.5)	.341
	**GB**	87 (9.2)	48 (12.2)	19 (17.8)	.004
	*P*-value	.631	<.001	.542	–
Copper deficiency	**SG**	65 (3.7)	26 (5.8)	8 (8.5)	.002
	**GB**	41 (4.7)	22 (8.5)	11 (13.9)	<.001
	*P*-value	.211	.178	.257	–
Zinc deficiency	**SG**	32 (1.6)	9 (1.3)	0 (0.0)	.010
	**GB**	25 (2.5)	16 (3.5)	10 (5.0)	.054
	*P*-value	.097	.011	<.001	–
Calcium deficiency	**SG**	32 (1.6%	4 (0.6%)	2 (1.8)	.246
	**GB**	23 (2.2)	16 (3.7%)	5 (4.2)	.121
	*P*-value	.173	<.001	.449	–
Phosphate deficiency	**SG**	32 (1.6)	2 (0.3)	0	.012
	**GB**	7 (0.7)	1 (0.2)	0	.187
	*P*-value	.040	>.999	–	–
Vitamin B12 deficiency	**SG**	251 (12.8)	78 (10.2)	8 (3.2	<.001
	**GB**	149 (14.9)	39 (8.3)	15 (7.6)	<.001
	*P*-value	.106	.265	.037	–
25 (OH) vitamin D deficiency	**SG**	997 (50.6)	140 (19.3)	27 (22.5)	<.001
	**GB**	484 (49.9)	83 (19.2)	23 (17.7)	<.001
	*P*-value	.726	.982	.342	–
Hypo albuminemia, < 2.5 g/dL	**SG**	0 (0.0)	0 (0.0)	0 (0.0)	–
	**GB**	0 (0.0)	11 (1.5)	3 (1.0)	0.003
	*P*-value	–	< 0.001	0.060	–

*SG* sleeve gastrectomy, *GB* gastric bypass

Data are presented as n (%)

#### Complications

The mean length of hospital stay was 2.5 days in both surgery groups. The mean surgery time and anesthesia duration were significantly shorter in the SG group, compared to the GB group (56.5 ± 16.2 and 105.2 ± 22.9 vs. 72.7 ± 24.3 and 121.8 ± 30.4 min, respectively; *P* < 0.001). Early and late complications are summarized in Table 4. There was only one case of early mortality in the GB group, while two and four late mortalities were reported in the SG and GB groups, respectively. Overall, 122 patients returned to the operating room, including 55 (2.4%) patients in the SG group and 67 (6.0%) patients in the GB group (*P* < 0.001). In general, the rate of early and late complications was lower in the SG group, compared to the GB group (4.6 and 2.2% vs. 11.7 and 5.2%, respectively; *P* < 0.001).

**Table 4:** Early and late complication in each surgery group

	SG (*n* = 2202)	GB (*n* = 1085)	*P*-value
Operation time, minutes	56.5 ± 16.2	72.7 ± 24.3	< 0.001
Anesthesia time, minutes	105.2 ± 22.9	121.8 ± 30.4	< 0.001
Hospital stay (range: 1–29), day	2.5 ± 2.8	2.5 ± 2.7	0.781
Early (30-day)	**103 (4.6%)**	**128 (11.7%)**	**< 0.001**
*Death*	0	1	–
*Re-operation*	**19 (0.8%)**	**39 (3.5%)**	**< 0.001**
Bleeding	10	21	–
Intestinal obstruction	1	5	–
Abscess/infection	5	4	–
Staple line leak	3	8	–
Marginal ulcer perforation	0	1	–
*Re-admission*	**25 (1.1%)**	**22 (2%)**	**0.043**
Infection/Fever	6	8	–
Deep vein thrombosis	8	4	–
Vomiting or poor intake	10	7	–
Marginal ulcer	0	2	–
GERD	1	1	–
*Hospital stay ≥ 7 day*	**59 (2.6%)**	**66 (6%)**	**< 0.001**
Infection/ Fever	8	4	–
Fluid or electrolyte depletion	4	1	–
Bleeding requiring transfusion	28	43	–
Pulmonary embolism	3	9	–
Pneumonia	7	4	–
Other	9	5	–
Late (> 30 days up to one year)	**49 (2.2%)**	**57 (5.2%)**	**< 0.001**
*Death*	**2**	**4**	**0.097**
Liver failure^a^	0	1	–
Myocardial infarction	0	1	–
Cancer	2	1	–
Other	0	1	–
*Re-operation*	**36 (1.6%)**	**28 (2.5%)**	**0.066**
Anastomosis stricture	0	2	–
Marginal ulcer perforation	0	1	–
Internal Hernia	1	2	–
Intestinal obstruction	0	1	–
Cholecystectomy	32	10	–
PCM needed revision surgery	0	7	–
Other	3	5	–
*Re-admission*	**11 (0.5%)**	**25 (2.3%)**	**< 0.001**
GERD	9	11	–
Wound Infection	2	2	–
PCM needed TPN	0	12	–

*SG* sleeve gastrectomy, *GB* gastric bypass, *GERD* Gasteroesophagial reflux disease, *PCM* protein calorie malnutrition, *TPN* Total Parenteral nutrition

^a^liver failure due to protein calorie malnutrition

#### Quality of life

A total of 560 patients were asked to complete SF-36 before and 12 months after surgery. Of these patients, 372 (66.4%) and 188 (33.6%) underwent SG and GB, respectively. There was no significant difference between the two groups in terms of physical and mental health components of quality of life at baseline and 12 months after surgery (Table 5). However, both groups improved significantly in all subdomains of quality of life after the operation (Figure 3 in the Supplementary Appendix).

**Table 5:** Quality of life subdomains scores and its alteration during 1 year after the operation in 560 patients

		Baseline	12th month	*p*-value
Physical Health Components				
Physical Functioning	SG	59.5 26.2	89.7 15.7	<.001
	GB	58.5 25.0	89.4 14.3	<.001
	*p*-value	.665	.918	–
Role Limitations due to Physical Health	SG	68.1 29.2	78.2 33.9	<.001
	GB	69.7 30.5	78.4 33.9	0.003
	*p*-value	.563	.926	–
Bodily Pain	SG	58.2 26.8	81.5 23.0	<.001
	GB	56.0 26.3	83.4 21.3	<.001
	*p*-value	.363	.181	–
General Health	SG	45.4 18.8	71.5 18.7	<.001
	GB	44.0 18.0	72.8 18.4	<.001
	*p*-value	.409	0.418	–
Mental Health Components				
Role Limitations due to Emotional Problems	SG	78.8 33.7	89.8 21.2	<.001
	GB	84.3 31.1	90.0 20.9	.009
	*p*-value	.062	.545	–
Energy/Fatigue	SG	45.0 24.5	58.2 22.0	<.001
	GB	44.5 22.8	59.2 21.4	<.001
	*p*-value	.824	.433	–
Emotional Well Being	SG	59.7 21.2	66.1 19.2	<.001
	GB	59.7 21.9	66.9 20.2	<.001
	*p*-value	.992	.539	–
Social Functioning	SG	56.2 26.8	78.6 21.7	<.001
	GB	55.9 27.7	79.5 21.9	<.001
	*p*-value	.913	.584	–

## Discussion

The current study is a comprehensive two-year report of two common bariatric procedures, comparing their effectiveness and safety. With respect to weight loss outcomes, GB was found to be slightly more beneficial than SG, although both procedures were efficiently successful. On the other hand, concerning the remission and improvement of obesity-associated comorbidities, both types of surgeries showed promising results. However, GB was more effective than SG in the remission of comorbidities, including HTN and dyslipidemia. In terms of T2DM remission, none of the surgical techniques were considered to be superior. Moreover, early and late surgical complications, as well as micronutrient deficiencies, were less prevalent in the SG group, compared to the GB group. Nonetheless, both surgeries played a beneficial role in improving the patients’ quality of life.

Previous meta-analyses have reported that both SG and GB, as the most popular bariatric procedures, have substantial effects on weight loss [14, 15]. However, several studies with longer follow-ups, including a meta-analysis of five RCTs with a five-year follow-up, reported that GB resulted in greater EWL% than SG [16–18]. According to the present study, both SG and GB resulted in a significant EWL%. However, GB had more prominent effects on EWL% and fat percentage reduction, compared to SG at 12- and 24-month follow-ups.

The remission and improvement of obesity-associated comorbidities are among other major goals of bariatric surgery. According to previous reports, different types of bariatric surgery are successful in resolving comorbidities, such as DM, HTN, and dyslipidemia [19, 20]. In this study, both SG and GB showed significant results regarding the remission and improvement of the mentioned comorbidities in all follow-ups. Based on the findings, T2DM remission and improvement were comparable between the SG and GB groups. In agreement with the present study, a recent meta-analysis of 11 RCTs, with 1–60 months of follow-up, showed that these two types of surgery were equivalent with respect to T2DM remission [21]. Additionally, several meta-analyses have reported similar results regarding T2DM remission and/or improvement [22–25].

Furthermore, according to the present study, HTN remission was comparable between the SG and GB groups, except for the 24-month follow-up, when GB was the superior procedure. This may indicate the more sustainable effect of GB on HTN remission in long-term follow-ups. Consistently, Climent et al. [26], in a meta-analysis, showed that HTN remission rate was higher in the GB group in both 1- and 5-year follow-ups. Moreover, we found that GB was a superior procedure regarding dyslipidemia remission in all follow-ups. Consistently, several studies reported that GB resulted in a higher rate of dyslipidemia remission in comparison with SG [6, 27].

Previous studies have shown that post-bariatric patients are prone to micronutrient deficiencies, such as vitamin D, folate, and vitamin B12 deficiencies due to the malabsorptive nature of this procedure [28–30]. Moreover, the GB procedure was found to be more prominent in causing nutritional deficiencies [31, 32]. In this regard, Enani et al. [33], in a recent meta-analysis, reported that iron deficiency is a common complication after bariatric surgeries, especially after GB surgery. Consistently, we found that vitamin D, vitamin B12, hemoglobin, and serum iron deficiencies were prevalent in both surgery groups, especially in the GB group.

Selection of either SG or GB does not solely depend on the effectiveness of the procedure, and safety plays an essential role, as well. In the current study, SG had more favorable outcomes regarding surgery duration and early complications. Consistently, Zhao et al. [34], in a recent meta-analysis, reported that SG is a superior surgical procedure to GB regarding the surgery duration and early complications. Similarly, another meta-analysis reported that SG was associated with fewer early complications (major and minor) [35]. On the other hand, late complications were comparable between the two procedures [36]. In this study, with respect to late complications, mortality and reoperation were not significantly different between the SG and GB groups; however, the readmission rate was lower in the SG group. Of note, gasteroesophageal reflux disease (GERD) is a considerable complication of obesity and bariatric surgery [37] and affected individuals experience discomfort, pain and emotional distress [38]. Similar to previous studies [39], further analysis showed that SG is a more favorable bariatric procedure in comparison with GB regarding GERD.

Quality of life improvement is another crucial aspect of bariatric surgery. Rausa et al. [40], in a recent meta-analysis, reported that both SG and GB could significantly improve the quality of life of patients. Moreover, Schauer et al. [41], in an RCT, found that both surgeries resulted in the significant improvement of quality of life during 5 years of follow-up. Similarly, we revealed that quality of life increased considerably in both physical and mental health domains 1 year after surgery, regardless of the type of surgery.

To the best of our knowledge, this is the first comprehensive report of two popular bariatric surgeries in the Middle East and MENA region. All major outcomes were compared between SG and GB. The patients underwent bariatric surgery and were followed-up in one center by a medical team. On the other hand, this study had some limitations. First, the patients were not randomized to the SG and GB groups; however, we attempted to compensate for this bias in the multivariable analyses by measuring PS for each patient. Second, the follow-up period was considered short for bariatric surgery. Lastly, the GB group consisted of both RYGB and OAGB patients.

## Conclusion

In conclusion, bariatric surgery is an effective and durable treatment for obesity, which can also alleviate obesity-associated comorbidities. SG and GB are the most popular bariatric surgeries worldwide. According to the findings of the present study, both SG and GB are effective in terms of weight loss, remission of obesity-associated comorbidities, and quality of life improvement. Since SG is associated with fewer complications and nutritional deficiencies, it can be considered a valid treatment for obesity and its associated comorbidities in eligible patients. However, further RCTs, with comorbidity remission as the primary endpoint, are needed to shed more light on the existing discrepancies regarding the decision to choose between different bariatric surgeries, especially SG and GB.

## Supplementary information

**Additional file 1: Table S1.** Nutritional Reference values.

**Additional file 2: Table S2.** Obesity related comorbidities remission and improvement definition.

**Additional file 3: Figure S1.** Anthropometric and body composition outcomes over time. **A**- BMI change (kg/m2) **B-** TWL (%). **C-** WC (cm). **D-** Lean mass (kg).

**Additional file 4: Figure S2.** Metabolic indices outcomes over time. **A-** FBS (mg/dL). **B-** HbA1c (%). **C-** TC (mg/dL). **D-** TG (mg/dL). **E-** LDL-C (mg/dL). **F-** HDL-C(mg/dL). **G-**SBP (mmHg). **H-** DBP (mmHg).

**Additional file 5: Figure S3.** Quality of life domains of the Iranian version of SF-36: A) The scores of patients in the SG group; and B) the scores of patients in the GB group.

## Data Availability

The datasets used and analyzed during the current study are available from the corresponding author on reasonable request.

## Abbreviation

SG: Sleeve gastrectomy
GB: gastric bypass
T2DM: Type 2 diabetes mellitus
RYGB: Roux-en-Y gastric bypass
EWL%: Excess weight loss
RCTs: Randomized clinical trials
MENA: Middle East
TOTS: Tehran Obesity Treatment Study
OAGB: One-anastomosis gastric bypass
FM: Fat mass
FFM: Fat-free mass
%FM: Percent body fat mass
FPG: Fasting plasma glucose
TG: Serum triglyceride
TC: Total cholesterol
HDL-C: High-density lipoprotein-cholesterol
∆BMI: Body mass index change
HTN: Hypertension
SF-36: Short-Form Health Survey
PS: Propensity score
SBP: Systolic blood pressure
DBP: Diastolic blood pressure
IPTW: Inverse probability of treatment weighting
GERD: Gasteroesophageal reflux disease
