# Supplementary material for: Two-year outcomes of sleeve gastrectomy versus gastric bypass: first report based on Tehran obesity treatment study (TOTS)
Source: BMC Surg. 2020 Jul 20;20:160. doi: 10.1186/s12893-020-00819-3 (PMC7370506; doi:10.1186/s12893-020-00819-3)
Supplement: Supplementary file 1 — Additional file 1: Table S1. Nutritional Reference values. [file 12893_2020_819_MOESM1_ESM.docx]

| Supp Table 1.Nutritional Reference values | | |
| --- | --- | --- |
|  | **Male** | **Female** |
| Hemoglobin (g/dl) | 14–17.5 | 12.3–15.3 |
| Hematocrit (%) | 41.5–50.4 | 35.9–44.6 |
| Total iron binding capacity (μg/dl) | 230–440 | 230–440 |
| Ferritin (ng/ml) | 15–200 | 12–150 |
| Iron (μg/dl) | 40–170 | 37–165 |
| Copper (μg/dl) | 70–140 | 80–155 |
| Zinc (μg/dl) | 50–150 | 50–150 |
| Calcium (mg/dl) | 8.5–10.5 | 8.5–10.5 |
| Phosphate (mg/dl) | 2.6–4.5 | 2.6–4.5 |
| Vitamin B12 (pmol/l) | 160–950 | 160–950 |
| 25 (OH) vitamin D (ng/ml) | ≥ 20 | ≥ 20 |
| Deficiencies were defined based on the amounts below the low limit of normal values | | |
