# Supplementary material for: Two-year outcomes of sleeve gastrectomy versus gastric bypass: first report based on Tehran obesity treatment study (TOTS)
Source: BMC Surg. 2020 Jul 20;20:160. doi: 10.1186/s12893-020-00819-3 (PMC7370506; doi:10.1186/s12893-020-00819-3)
Supplement: Supplementary file 2 — Additional file 2: Table S2. Obesity related comorbidities remission and improvement definition. [file 12893_2020_819_MOESM2_ESM.docx]

| Supp Table 2. Obesity related comorbidities remission and improvement definition | | |
| --- | --- | --- |
| Diabetes | remission | (HbA1c < 6.5%, FBG < 126 mg/dL) in the absence antidiabetic medications |
|  | Improvement | Statistically significant reduction in HbA1c and FBG not meeting criteria for remission or decrease in antidiabetic medications requirement (by discontinuing insulin or one oral agent, or half reduction in dose) |
| Hypertension | remission | Defined as SBP<140 mmHg and DBP<90 mmHg off antihypertensive medication. |
|  | Improvement | Defined as a decrease in number of antihypertensive medication or decrease in systolic or diastolic blood pressure (BP) on the same medication |
| Dyslipidemia | Remission | Normal lipid panel off medication. (LDL<100 mg/dL and HDL<40 mg/dL and total cholesterol<200 mg/dL and Triglyceride<150 mg/dL. |
|  | Improvement | Decrease in number of lipid-lowering agents with equivalent control of dyslipidemia or improved control of lipids on equivalent medication. |
