# Supplementary material for: Two-year outcomes of sleeve gastrectomy versus gastric bypass: first report based on Tehran obesity treatment study (TOTS)
Source: BMC Surg. 2020 Jul 20;20:160. doi: 10.1186/s12893-020-00819-3 (PMC7370506; doi:10.1186/s12893-020-00819-3)
Supplement: Supplementary file 3 — Additional file 3: Figure S1. Anthropometric and body composition outcomes over time. A- BMI change (kg/m2) B- TWL (%). C- WC (cm). D- Lean mass (kg). [file 12893_2020_819_MOESM3_ESM.docx]

**Supp Figure1.**
