# Supplementary material for: Two-year outcomes of sleeve gastrectomy versus gastric bypass: first report based on Tehran obesity treatment study (TOTS)
Source: BMC Surg. 2020 Jul 20;20:160. doi: 10.1186/s12893-020-00819-3 (PMC7370506; doi:10.1186/s12893-020-00819-3)
Supplement: Supplementary file 4 — Additional file 4: Figure S2. Metabolic indices outcomes over time. A- FBS (mg/dL). B- HbA1c (%). C- TC (mg/dL). D- TG (mg/dL). E- LDL-C (mg/dL). F- HDL-C(mg/dL). G-SBP (mmHg). H- DBP (mmHg). [file 12893_2020_819_MOESM4_ESM.docx]

**Supp Figure 2.**
