# Supplementary material for: Two-year outcomes of sleeve gastrectomy versus gastric bypass: first report based on Tehran obesity treatment study (TOTS)
Source: BMC Surg. 2020 Jul 20;20:160. doi: 10.1186/s12893-020-00819-3 (PMC7370506; doi:10.1186/s12893-020-00819-3)
Supplement: Supplementary file 5 — Additional file 5: Figure S3. Quality of life domains of the Iranian version of SF-36: A) The scores of patients in the SG group; and B) the scores of patients in the GB group. [file 12893_2020_819_MOESM5_ESM.docx]

**Supp Figure 3**

A-SG

B-GB
